# The Effect of Sodium-Glucose Cotransporter-2 (SGLT2) Inhibitors on Cardiovascular Outcomes in Cancer Patients With Type 2 Diabetes Mellitus: A Systematic Review

**DOI:** 10.7759/cureus.85220

**Published:** 2025-06-02

**Authors:** Sara Omer Hamid Zain Elabdin, Mudather Abdelgabar Ali Mohammed, Awab Salah Eldin Hassan Elsheikh, Myada Omer Hussien Hamed, Mona Omar Elgezoly Basher, Emad Salah Fathy Saleh

**Affiliations:** 1 Geriatric Medicine, Stepping Hill Hospital-Stockport NHS Foundation Trust, Stockport, GBR; 2 Anatomical Sciences, St. George’s University, St. George's, GRD; 3 Internal Medicine, Zayed Military Hospital, Abu Dhabi, ARE; 4 Internal Medicine, Andalusia Hospital, Jeddah, SAU; 5 Internal Medicine, Alqunfudah General Hospital, Alqunfudah, SAU; 6 Internal Medicine, Nizwa Hospital, Nizwa, OMN

**Keywords:** cancer, cardio-oncology, cardiovascular outcomes, sglt2 inhibitors, systematic review, type 2 diabetes

## Abstract

While the cardiovascular benefits of sodium-glucose cotransporter-2 inhibitors (SGLT2is) in patients with type 2 diabetes mellitus (T2DM) are well-established, their role in cancer patients with T2DM - a population at heightened risk of cardiovascular complications due to both malignancy and cardiotoxic therapies - remains unclear. This systematic review aimed to synthesize the existing evidence on the effects of SGLT2is on cardiovascular outcomes in this high-risk group. We conducted a comprehensive literature search across PubMed/MEDLINE, Embase, Scopus, and Web of Science following the Preferred Reporting Items for Systematic Reviews and Meta-Analyses (PRISMA) 2020 guidelines. Observational cohort studies and randomized controlled trials (RCTs) evaluating SGLT2is in adult cancer patients with T2DM were included. Data extraction and quality assessment (using the Newcastle-Ottawa Scale for cohort studies) were performed independently by two reviewers. Due to clinical and methodological heterogeneity, findings were synthesized narratively, with effect estimates [hazard ratios (HRs), odds ratios (ORs)] and 95% confidence intervals (CIs) reported.

Nine studies (n = 162,605 participants) were included, comprising retrospective cohorts (n = 7) and population-based studies (n = 2). SGLT2i use was consistently associated with reduced all-cause mortality and heart failure (HF)-related hospitalizations. Benefits were particularly pronounced in patients exposed to anthracyclines or with pre-existing cardiovascular risk. Subgroup analyses suggested a dose-dependent survival advantage, while safety outcomes were comparable to non-users. Study quality was generally high, though heterogeneity precluded meta-analysis. SGLT2 inhibitors appear to confer significant cardiovascular protection in cancer patients with T2DM, particularly against mortality and HF. These findings support their cautious integration into cardio-oncology practice, though randomized trials are needed to confirm causality and optimize protocols. Clinicians should weigh individual risks, especially in immunocompromised patients, while researchers should prioritize prospective studies to clarify mechanisms and long-term effects.

## Introduction and background

The intersection of cancer and cardiovascular disease represents one of the most clinically significant challenges in contemporary medicine, particularly for patients with comorbid type 2 diabetes mellitus (T2DM) [1]. Sodium-glucose cotransporter-2 inhibitors (SGLT2is), initially developed as glucose-lowering agents, have emerged as a therapeutic class with profound cardioprotective benefits. This has been demonstrated in major cardiovascular outcome trials such as EMPA-REG OUTCOME (Empagliflozin Cardiovascular Outcome Event Trial in Type 2 Diabetes Mellitus Patients - Removing Excess Glucose) [2] and DAPA-HF (Dapagliflozin and Prevention of Adverse Outcomes in Heart Failure) [3].

The EMPA-REG OUTCOME trial evaluated empagliflozin in patients with T2DM and established cardiovascular disease, showing a significant reduction in cardiovascular death and hospitalization for heart failure (HF) [2]. Similarly, the DAPA-HF trial assessed dapagliflozin in patients with HF with reduced ejection fraction, including those without diabetes, and found a substantial reduction in the risk of worsening HF or cardiovascular death [3]. These landmark trials have significantly influenced clinical guidelines. However, while the cardiovascular benefits of SGLT2 inhibitors in general populations with T2DM or HF are well-established [4], their role in cancer patients with T2DM remains poorly characterized, despite compelling biological plausibility and growing clinical need.

Cancer patients with T2DM represent a particularly vulnerable population, facing the dual burden of malignancy-related metabolic dysregulation and increased cardiovascular risk [5]. The pathophysiology underlying this risk is multifactorial, involving direct cardiotoxic effects of many cancer therapies, metabolic perturbations from both diabetes and malignancy, and systemic inflammation that accelerates vascular and myocardial dysfunction [6]. Anthracyclines, radiation therapy, and newer targeted agents are all known to cause cardiovascular complications ranging from HF to arrhythmias, while diabetes itself exacerbates these risks through mechanisms such as advanced glycation end-product accumulation and mitochondrial dysfunction [7]. This complex interplay creates a perfect storm of cardiovascular vulnerability that may be uniquely amenable to SGLT2is' pleiotropic effects [8].

The mechanistic rationale for SGLT2i use in this population is robust. Beyond their glucose-lowering effects, these agents have been shown to improve myocardial energetics, reduce systemic inflammation, attenuate oxidative stress, and promote favorable hemodynamic changes through natriuresis and reduced ventricular afterload [9]. Preclinical data suggest SGLT2is may specifically mitigate anthracycline-induced cardiotoxicity by preserving mitochondrial function and reducing cardiomyocyte apoptosis [10]. Furthermore, their potential to counteract the metabolic reprogramming characteristic of many malignancies adds another dimension to their therapeutic promise in oncology patients [11].

Despite this strong mechanistic foundation, the clinical evidence remains fragmented across observational studies of varying designs and quality. Several recent cohort studies have reported significant reductions in HF-related hospitalizations and mortality among cancer patients with T2DM receiving SGLT2 inhibitors, but these findings have not been systematically evaluated. The existing literature also shows important gaps in terms of understanding class effects, optimal timing of initiation relative to cancer therapy, and differential benefits across cancer types and treatment modalities. Moreover, safety considerations specific to this population, including potential interactions with cancer therapies and risks in immunocompromised states, require careful examination.

This systematic review, therefore, aims to synthesize the current evidence on cardiovascular outcomes associated with SGLT2i use in cancer patients with T2DM. By critically evaluating study methodologies, quantifying effect sizes where possible, and identifying knowledge gaps, we seek to inform clinical decision-making and guide future research directions. Our analysis is particularly timely given the expanding use of SGLT2is in cardiometabolic practice and the growing recognition of cardio-oncology as a distinct subspecialty. The findings will be relevant to oncologists, cardiologists, and endocrinologists managing this high-risk population, as well as to researchers exploring novel cardioprotective strategies in cancer patients.

## Review

Metodology

This systematic review was conducted per the Preferred Reporting Items for Systematic Reviews and Meta-Analyses (PRISMA) 2020 guidelines [12] to ensure methodological rigor, transparency, and reproducibility.

Eligibility Criteria

Studies were selected based on predefined inclusion and exclusion criteria. We included observational cohort studies (prospective or retrospective) and randomized controlled trials (RCTs) that evaluated the effects of SGLT2 inhibitors on cardiovascular outcomes in adult cancer patients (≥18 years) with T2DM. The primary outcomes of interest were cardiovascular mortality, hospitalization due to HF, and major adverse cardiovascular events (MACE). Secondary outcomes included all-cause mortality, arrhythmias, and renal outcomes. Studies not reporting comparative data between SGLT2i users and non-users, those involving non-human subjects, or those that were conference abstracts, reviews, or case reports with fewer than 10 participants. 

Information Sources and Search Strategy

A comprehensive literature search was performed across multiple electronic databases, including PubMed/MEDLINE, Embase, Scopus, and Web of Science. The search strategy combined Medical Subject Headings (MeSH) terms and free-text keywords related to SGLT2 inhibitors, cancer, cardiovascular outcomes, and diabetes. No language restrictions were applied, and non-English articles were translated where necessary. The detailed search strategy is provided in the supplementary file in the Appendices section.

Study Selection Process

Two independent reviewers (MOEB and ESFS) screened titles and abstracts manually. Full-text articles of potentially eligible studies were retrieved and assessed for inclusion based on the predefined criteria. Discrepancies between reviewers were resolved through discussion or consultation with a third reviewer (SOHZE), who served as a tiebreaker. The study selection process was documented in a PRISMA 2020 flow diagram, detailing the number of records identified, screened, excluded, and included, along with reasons for exclusion.

Data Extraction and Management

Two independent reviewers (MOEB and ESFS) performed data extraction using a standardized, pilot-tested form in Microsoft Excel (Microsoft Corporation, Redmond, WA) to ensure consistency and accuracy. The extracted variables encompassed study characteristics, participant demographics, intervention details, outcome measures, and key findings. Any disagreements between reviewers during the extraction process were resolved through discussion and consensus. In cases where data were missing or unclear, attempts were made to contact the original study authors to obtain the necessary information, thereby enhancing the completeness and reliability of the dataset. This rigorous approach to data extraction minimized potential biases and ensured the robustness of the systematic review's findings.

Risk of Bias Assessment

The methodological quality of the included cohort studies was systematically evaluated using the Newcastle-Ottawa Scale (NOS) [13], a validated tool designed to assess risk of bias in non-randomized studies. The NOS evaluates three key domains: selection of study groups, comparability of cohorts, and ascertainment of outcomes. In the selection domain (maximum 4 points), studies were assessed based on the representativeness of the exposed cohort, selection of non-exposed participants, ascertainment of exposure, and demonstration that outcomes were not present at baseline. The comparability domain (maximum 2 points) examined whether studies controlled for confounding variables through design or analysis, such as matching or statistical adjustment for critical factors like age, comorbidities, or cancer stage. Finally, the outcome domain (maximum 3 points) evaluated the methods of outcome assessment, follow-up duration, and adequacy of follow-up rates. Studies scoring 7-9 points were classified as low risk of bias, 5-6 as moderate risk, and ≤4 as high risk.

Data Synthesis and Analysis

Due to significant clinical and methodological heterogeneity observed across the included studies, which varied in terms of patient populations, cancer types, SGLT2 inhibitor regimens, and outcome definitions, a narrative synthesis approach was deemed most appropriate for this systematic review. This synthesis was structured by key outcome categories such as mortality and HF events, with effect estimates including hazard ratios (HRs) and odds ratios (ORs) along with their 95% confidence intervals (CIs) being systematically reported and compared across studies. While meta-analysis would have provided pooled effect estimates, the substantial variability in study designs and patient characteristics made meaningful statistical pooling unreliable. The narrative approach allowed for a more nuanced interpretation of findings while appropriately acknowledging the limitations imposed by the variability across studies.

Results

Study Selection Process

**Figure 1:**
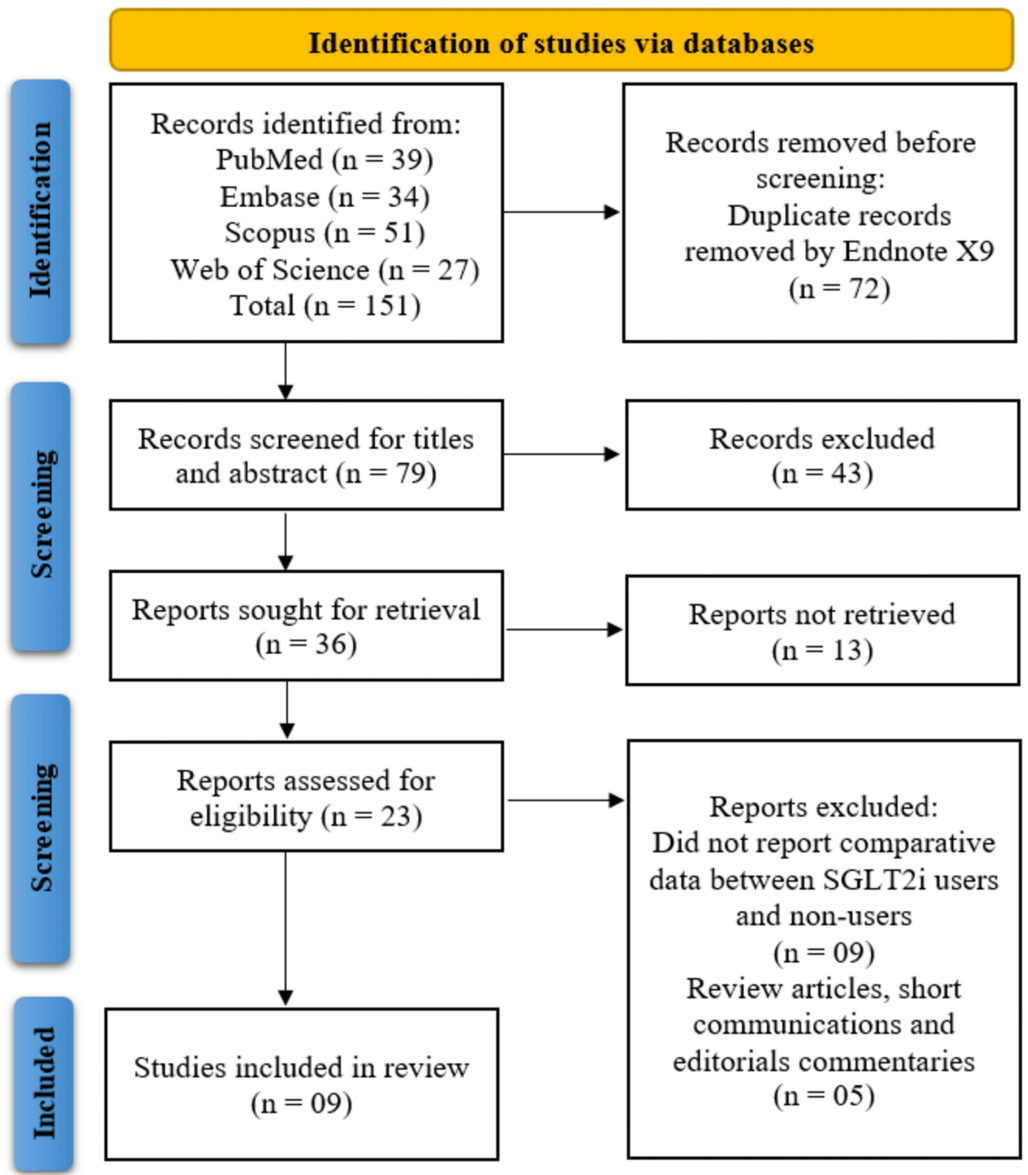
PRISMA flow diagram illustrating study selection PRISMA: Preferred Reporting Items for Systematic Reviews and Meta-Analyses; SGLT2i: sodium-glucose cotransporter-2 inhibitor

The systematic search across PubMed, Embase, Scopus, and Web of Science initially identified 151 records, from which 72 duplicates were removed using EndNote X9, leaving 79 unique records for title and abstract screening. Of these, 43 were excluded as irrelevant, and 36 full-text articles were sought for retrieval. Thirteen reports could not be retrieved, and a further 14 were excluded (nine for lacking comparative data between SGLT2 inhibitor users and non-users, and five as review articles, short communications, or editorials). Ultimately, 23 full-text articles were assessed for eligibility, with nine studies [14-22] meeting the inclusion criteria and being incorporated into the systematic review (Figure 1).

Study Characteristics

The included studies comprised observational and retrospective cohort designs, with sample sizes ranging from 119 to 81,572 participants. Studies were conducted across diverse geographic regions, including the USA, Canada, South Korea, Taiwan, and Israel (Table 1). The populations predominantly consisted of cancer patients with pre-existing T2DM, and the types of cancer varied, including hepatocellular carcinoma (HCC), non-small cell lung cancer (NSCLC), and others treated with cardiotoxic therapies such as anthracyclines or immune checkpoint inhibitors (ICIs). SGLT2is were compared to non-users or guideline-directed therapies without SGLT2is. Follow-up durations ranged from 1.5 years to seven years, with cardiovascular outcomes assessed as primary or secondary endpoints.

**Table 1:** Characteristics of included studies HCC: hepatocellular carcinoma; T2DM: type 2 diabetes mellitus; SGLT2i: sodium-glucose co-transporter 2 inhibitor; NR: not reported; HF: heart failure; HR: hazard ratio; CI: confidence interval; CTRCD: cancer therapy-related cardiac dysfunction; ED: emergency department; AKI: acute kidney injury; NSCLC: non-small cell lung cancer; CVD: cardiovascular disease; MI: myocardial infarction; DM: diabetes mellitus; AC: anthracycline; aHR: adjusted hazard ratio; ICIs: immune checkpoint inhibitors; MACE: major adverse cardiovascular events

Study	Country	Study design	Sample size	Population characteristics	Type of cancer	SGLT2 inhibitor used	Comparator	Duration of follow-up	Cardiovascular outcomes assessed	Main findings
Hendryx et al., 2022 [14]	USA	Observational cohort	3,185	HCC patients ≥66 years with pre-existing T2DM diagnosed 2014–2017	Hepatocellular carcinoma (HCC)	SGLT2 inhibitors (unspecified types)	No SGLT2 inhibitor use	Up to the end of 2019 (median not specified)	Overall mortality (as proxy for CV risk)	SGLT2i use was associated with significantly reduced mortality risk (HR = 0.68; CI = 0.54–0.86); stronger benefit with longer use (HR = 0.60; CI = 0.41–0.88)
Gongora et al., 2022 [15]	USA	Retrospective cohort	128 (32 cases, 96 controls)	Patients with diabetes mellitus and cancer treated with anthracyclines	NR	NR	No SGLT2 inhibitor (matched controls)	Median 1.5 years	Composite of heart failure incidence, HF admissions, new cardiomyopathy, arrhythmias	SGLT2i use was linked to lower cardiac events (3% vs 20%), lower mortality (9% vs 43%), and lower sepsis/neutropenic fever (16% vs 40%)
Avula et al., 2024 [16]	USA	Retrospective cohort study	1,280 (640 SGLT2i users, 640 controls)	Adults (≥18 years) with T2DM, cancer, prior exposure to cardiotoxic cancer therapy, and subsequent CTRCD or HF; mean age 67.6; 41.6% female; 68% White	Any cancer treated with cardiotoxic therapies)	SGLT2 inhibitors (unspecified types)	Guideline-directed medical therapy without SGLT2 inhibitors	2 years	Acute HF exacerbation, all-cause mortality, all-cause hospitalizations/ED visits, atrial fibrillation/flutter, acute kidney injury, renal replacement therapy	SGLT2 inhibitor use significantly reduced the risk of acute HF, mortality, hospitalizations, atrial fibrillation, AKI, and need for dialysis compared to controls
Chiang et al., 2023 [17]	USA	Retrospective propensity score-matched cohort study	878 SGLT2i recipients (matched to 878 controls, total n=1756)	Adults with T2DM diagnosed with cancer between 2010 and 2021	NR	SGLT2 inhibitors (unspecified type)	Non-recipients of SGLT2i	Median 18.8 months	Hospitalisation for incident HF, All-cause mortality	72% reduction in HF hospitalization risk; higher 2-year overall survival (85.3% vs 63.0%); no difference in adverse events
Luo et al., 2023 [18]	USA	Retrospective cohort (observational)	24,915	NSCLC patients aged ≥66 with pre-existing diabetes	Non-small cell lung cancer (NSCLC)	NR	Non-users of SGLT2 inhibitors	Up to ~5 years (2014–2019)	Overall mortality (as a proxy for CV outcome)	SGLT2i use significantly reduced mortality (HR = 0.68); stronger effect with longer use (HR = 0.54)
Abdel-Qadir et al., 2023 [19]	Canada	Population-based cohort study	933	Patients >65 years with treated T2DM, no prior HF, received anthracyclines	NR	SGLT2i (unspecified)	Non-SGLT2i controls	Median 1.6 years	HF hospitalization, incident HF diagnosis, any CVD diagnosis, mortality	SGLT2i associated with reduced HF hospitalization (HR 0, p<0.001); no sig. difference in other CV outcomes or mortality
Hwang et al., 2023 [20]	South Korea	Retrospective cohort study (emulated target trial)	81,572	Patients undergoing anthracycline (AC)-based chemotherapy, 780 with T2DM using SGLT2i	NR	NR	Non-SGLT2i hypoglycemic agents; non-DM group	2014–2021 (up to 7 years)	Composite of heart failure hospitalization, acute MI, ischemic stroke, and death	SGLT2i use in T2DM patients was associated with significantly better cardiovascular outcomes compared to non-DM and non-SGLT2i users (adjusted HR = 0.35 vs non-DM; 0.47 vs non-SGLT2i)
Huang et al., 2024 [21]	Taiwan	Propensity Score–Matched Cohort Study	50,133 (16,711 SGLT2i users; 33,422 non-users)	Patients with T2DM and cancer undergoing standard curative treatments	Multiple cancers (not specified)	SGLT2 inhibitors (unspecified type)	Non-users of SGLT2is	NR	NR	SGLT2i use was associated with significantly lower cancer-specific (aHR: 0.21) and all-cause mortality (aHR: 0.22); survival improved dose-dependently
Perelman et al., 2024 [22]	Israel	Retrospective	119	Cancer patients with type 2 diabetes mellitus (DM2); baseline cardiac risk factors comparable; higher ischemic heart disease in the SGLT2i group	Various cancers treated with ICIs	NR	No SGLT2i treatment	Median 28 months	MACE: myocarditis, acute coronary syndrome, heart failure, arrhythmia; all-cause mortality	SGLT2i was associated with significantly lower all-cause mortality (21% vs 59%, p=0.002); no myocarditis or atrial fibrillation cases in the SGLT2i group

Cardiovascular Outcomes Associated With SGLT2 Inhibitor Use** **

1. Reduction in mortality risk: Several studies demonstrated a significant reduction in all-cause mortality among cancer patients with T2DM treated with SGLT2is. Hendryx et al. [14] reported an HR of 0.68 (95% CI: 0.54-0.86) for overall survival in HCC patients, with stronger benefits observed with prolonged use (HR = 0.60; 95% CI: 0.41-0.88). Similarly, Luo et al. [18] found a 32% reduction in mortality risk (HR = 0.68; 95% CI: 0.60-0.77) among NSCLC patients, while Huang et al. [21] reported an even more pronounced reduction (adjusted HR = 0.22; 95% CI: 0.21-0.23) in a large cohort of cancer patients. Perelman et al. [22] also noted significantly lower all-cause mortality in SGLT2i users (21% vs. 59%, p = 0.002). 

2. Heart failure and hospitalization outcomes: SGLT2 inhibitors were consistently associated with reduced risks of HF hospitalization and composite cardiovascular events. Chiang et al. [17] observed a 72% reduction in HF hospitalization risk (HR = 0.28; 95% CI: 0.11-0.77), while Hwang et al. [20] reported a 65% lower risk of composite cardiovascular outcomes (HR = 0.35; 95% CI: 0.25-0.51) in anthracycline-treated patients. Avula et al. [16] further supported these findings, showing significant risk reductions for acute HF exacerbations, hospitalizations, and atrial fibrillation (ORs ranging from 0.296 to 0.486; p < 0.05). 

3. Composite cardiovascular events: Studies evaluating composite endpoints, including HF, acute myocardial infarction, stroke, and mortality, demonstrated favorable outcomes with SGLT2i use. Gongora et al. [15] reported lower cardiac event rates (3% vs. 20%) and mortality (9% vs. 43%) in anthracycline-treated patients. Abdel-Qadir et al. [19] found reduced HF hospitalization risk (HR = 0.39; 95% CI: 0.12-1.28), though other cardiovascular outcomes did not reach statistical significance. 

Statistical and Methodological Considerations

Effect estimates were predominantly derived from Cox proportional hazards models, with most studies adjusting for confounders such as demographics, tumor characteristics, and baseline cardiovascular risk factors (Table 2). Propensity score matching was frequently employed to address potential biases. Despite variability in outcome definitions and cancer types, the consistency in direction and magnitude of effect estimates across studies strengthens the evidence for cardiovascular benefits of SGLT2is in this population. 

**Table 2:** Cardiovascular Outcome Measures and Effect Estimates HCC: hepatocellular carcinoma; T2DM: type 2 diabetes mellitus; HR: hazard ratio; OR: odds ratio; CI: confidence interval; NR: not reported; HF: heart failure; AF: atrial fibrillation; AKI: acute kidney injury; CTRCD: cancer therapy-related cardiac dysfunction; NSCLC: non-small cell lung cancer; ATT: average treatment effect on the treated; DM: diabetes mellitus; aHR: adjusted hazard ratio.​​​​​​

Study	Outcome measure	Definition of outcome	Statistical method	Effect estimate (e.g., HR, OR)	95% CI	P-value	Adjusted confounders
Hendryx et al., 2022 [14]	Overall survival	Mortality risk among HCC patients with T2DM	Cox proportional hazards model (implied)	HR = 0.68	0.54 – 0.86	NR	Patient demographics, tumor characteristics, cancer treatments
Gongora et al., 2022 [15]	Cardiac events	Composite of HF incidence, admissions, new cardiomyopathy, and arrhythmias	NR	NR	NR	0.025	Age, sex, ethnicity, cancer type, cancer stage, cardiac risk factors
Avula et al., 2024 [16]	Composite of cardiovascular events and mortality	Acute HF exacerbation, all-cause mortality, hospitalizations, AF/flutter, AKI, renal replacement in CTRCD/HF patients with T2DM and cancer	ORs and Cox proportional HRs after propensity score matching	ORs ranged from 0.296 to 0.486, indicating risk reduction with SGLT2 inhibitors	95% CIs ranged approximately 0.19 –0.74	All p-values <0.05 (mostly <0.001)	Propensity score matching (adjusted for baseline confounders)
Chiang et al., 2023 [17]	Hospitalisation for HF	Incident hospitalization due to heart failure	Cox regression	HR = 0.28	0.11 – 0.77	0.013	Propensity score matching
Luo et al., 2023 [18]	Overall mortality risk	Death from any cause among NSCLC patients with T2DM	Cox proportional hazards	HR = 0.68	0.60 – 0.77	NR	Demographics, tumor characteristics, cancer treatments, and other potential confounders
Abdel-Qadir et al., 2023 [19]	Cardiovascular disease diagnosis	Hospitalization with documented cardiovascular disease	Cause-specific Cox model	HR = 0.39	0.12 – 1.28	0.12	Adjusted using propensity scores and ATT weighting
Hwang et al., 2023 [20]	Composite cardiovascular outcome	Heart failure hospitalization, acute myocardial infarction, ischemic stroke, and death	Cox proportional hazards model (after propensity score matching)	HR = 0.35 (vs. non-DM group)	0.25 – 0.51	NR	Propensity score matched (demographics, clinical characteristics)
Huang et al., 2024 [21]	All-cause mortality	Death due to any cause among cancer patients with T2DM	Cox proportional hazards regression	aHR = 0.22	0.21 – 0.23	<0.001	Propensity score matched covariates
Perelman et al., 2024 [22]	All-cause mortality	Death from any cause during the follow-up period	Comparison of mortality rates (retrospective analysis)	NR	NR	0.002	NR

Risk of Bias Assessment

**Figure 2:**
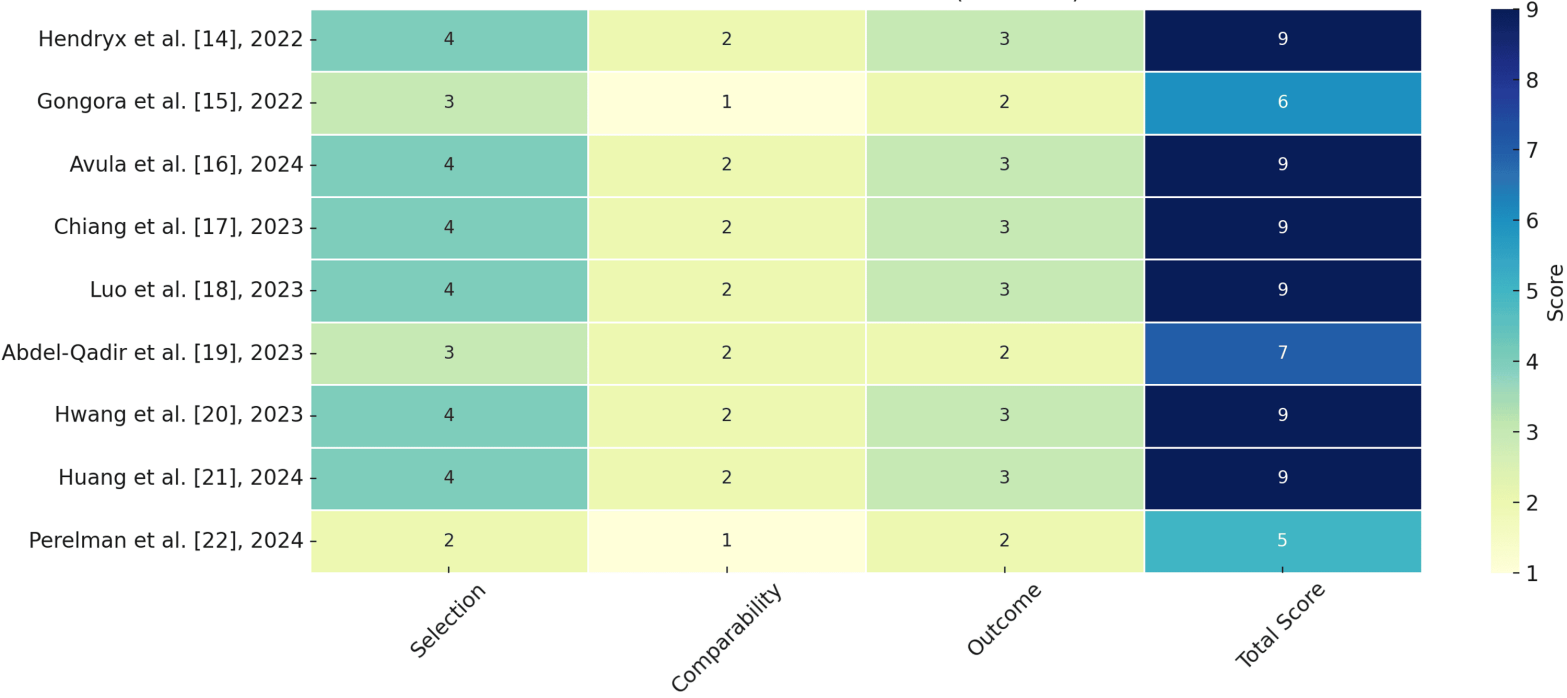
Heatmap of risk of bias assessment using the Newcastle-Ottawa Scale (NOS) The scores of individual studies across three NOS domains—Selection (maximum 4 points), Comparability (maximum 2 points), and Outcome (maximum 3 points)—with a total possible score of 9. Higher scores indicate lower risk of bias. Study labels include author names, citation numbers, and publication years. Color intensity corresponds to the magnitude of the score in each domain, facilitating visual comparison of methodological quality across included studies

The majority of studies demonstrated low risk of bias, with six out of nine [14,16-18,20,21] achieving the maximum NOS score of 9, reflecting rigorous methodology in cohort selection, robust adjustment for confounders, and reliable outcome measurement. Abdel-Qadir et al. [19] scored 7, indicating generally low risk of bias with minor limitations in outcome assessment. Two studies exhibited moderate risk: Gongora et al. [15] (6/9) due to a smaller sample size and limited confounder adjustment, and Perelman et al. [22] (5/9) because of incomplete follow-up data and a restricted cohort. The predominance of high-quality studies strengthens confidence in the review’s findings, while results from moderate-risk studies warrant cautious interpretation (Figure 2).

Discussion

The findings of this systematic review highlight a consistent association between SGLT2i use and improved cardiovascular outcomes in cancer patients with T2DM. The pooled evidence from observational and retrospective cohort studies suggests that SGLT2 inhibitors confer significant reductions in mortality, HF hospitalizations, and composite cardiovascular events, even in populations exposed to cardiotoxic cancer therapies such as anthracyclines or immune checkpoint inhibitors. These results align with the well-established cardioprotective effects of SGLT2is in non-cancer populations, as demonstrated in large cardiovascular outcome trials (CVOTs) such as EMPA-REG OUTCOME [2], DECLARE-TIMI 58 [23], and DAPA-HF [3]. However, the unique context of cancer and its treatment introduces additional complexities, including chemotherapy-induced cardiotoxicity, metabolic derangements, and immune-related cardiovascular complications, which may modulate the effects of SGLT2is. 

One of the most striking observations across the included studies was the reduction in all-cause mortality among SGLT2i users, with hazard ratios (HRs) ranging from 0.22 to 0.68 [14,18,21]. These findings are particularly noteworthy given the high baseline mortality risk in cancer patients, especially those with pre-existing T2DM. The mechanisms underlying this survival benefit are likely multifactorial. Beyond their glucose-lowering effects, SGLT2is have been shown to improve hemodynamic load, reduce systemic inflammation, and mitigate oxidative stress - all of which are pathways implicated in both cancer progression and cardiovascular disease (CVD) [24]. Additionally, SGLT2 inhibitors may attenuate cancer therapy-related cardiac dysfunction (CTRCD), a common complication of anthracyclines and targeted therapies, by preserving myocardial energetics and reducing fibrosis [15,16]. The dose-dependent survival benefit observed in some studies [21] further supports a causal relationship, though residual confounding cannot be entirely ruled out due to the observational nature of the data. 

The reduction in HF hospitalizations and composite cardiovascular events was another consistent finding, with HRs as low as 0.28 for incident HF [17] and 0.35 for major adverse cardiovascular events (Hwang et al., 2023). These results mirror those from non-cancer CVOTs, where SGLT2 inhibitors reduced HF hospitalizations by approximately 30-35% [2,3]. However, the magnitude of benefit in cancer patients appears even more pronounced, possibly due to the heightened cardiovascular risk in this population. For instance, anthracycline-induced cardiomyopathy is characterized by early diastolic dysfunction and late-onset HF, which may be particularly amenable to SGLT2i-mediated improvements in ventricular loading conditions and metabolic substrate utilization [15,19]. Interestingly, the study by Avula et al. [16] extended these findings to patients with established CTRCD or HF, demonstrating that SGLT2 inhibitors reduced not only HF exacerbations but also atrial fibrillation and acute kidney injury. This suggests a class-wide pleiotropic effect that extends beyond HF prevention to broader cardiorenal protection, a phenomenon well-documented in non-cancer populations [25]. 

Despite these encouraging results, the heterogeneity in study designs, cancer types, and outcome definitions complicates direct comparisons. For example, while most studies focused on solid tumors (e.g., HCC, NSCLC), others included heterogeneous cancer populations or specific treatment exposures (e.g., anthracyclines, ICIs) [20,22]. This variability may explain the differing effect sizes for certain outcomes, such as the non-significant reduction in non-HF cardiovascular events reported by Abdel-Qadir et al. [19]. Additionally, the lack of granular data on SGLT2i subtypes (e.g., empagliflozin vs. dapagliflozin) limits insights into potential drug-specific effects, though current evidence suggests class-wide benefits [26]. 

The observed cardiovascular benefits of SGLT2 inhibitors in cancer patients also raise important questions about underlying mechanisms. Preclinical studies suggest that SGLT2is may mitigate doxorubicin-induced cardiotoxicity by upregulating autophagy and reducing mitochondrial dysfunction [27]. Similarly, their anti-inflammatory properties could counteract the pro-inflammatory state induced by immune checkpoint inhibitors, which is increasingly recognized as a driver of myocarditis and arrhythmias [22]. These mechanistic insights, though preliminary, provide a plausible biological basis for the clinical findings and warrant further investigation in translational studies. 

Comparisons with existing literature on SGLT2is in non-cancer populations reveal both consistencies and unique considerations. The mortality and HF benefits align closely with those seen in CVOTs, reinforcing the class’s cardioprotective profile. However, cancer patients often exhibit distinct risk factors, such as cachexia, chronic inflammation, and high oxidative stress, which may amplify the relative benefits of SGLT2is [18]. Conversely, concerns about volume depletion and ketoacidosis-known adverse effects of SGLT2is-may be heightened in cancer patients due to chemotherapy-induced nausea, vomiting, and malnutrition. Reassuringly, none of the included studies reported significant safety signals, though dedicated pharmacovigilance studies are needed to confirm this. 

Limitations

This study has several limitations. Firstly, all included studies were observational, leaving them vulnerable to residual confounding despite adjustments and propensity score matching. Unmeasured variables, such as socioeconomic status, adherence to other medications, or cancer treatment modifications, could influence outcomes. Second, the predominance of retrospective designs introduces risks of selection and information bias, particularly in outcome ascertainment. Third, the generalizability of findings may be limited by the underrepresentation of certain cancer types (e.g., hematologic malignancies) and ethnic groups. Fourth, the lack of RCTs precludes definitive causal inferences, though the consistency of results across studies strengthens the evidence. Finally, the variability in follow-up durations and outcome definitions complicates cross-study comparisons.

## Conclusions

The consistent reductions in mortality, HF-related hospitalizations, and composite cardiovascular events across diverse cancer populations and treatment settings suggest a class-wide benefit from SGLT2is, which mirrors findings in non-cancer cohorts. While the observational nature of the data necessitates cautious interpretation, the mechanistic plausibility and magnitude of effect sizes support the consideration of SGLT2is as adjunctive therapy in high-risk patients. Further RCTs are urgently needed to confirm these findings and elucidate optimal patient selection, dosing, and safety monitoring protocols in this vulnerable population. Until then, the current evidence supports a favorable risk-benefit profile for SGLT2is in oncology patients with T2DM and high cardiovascular risk.

## Appendices

**Table 3:** Search strategy

Database	Search string
PubMed/MEDLINE	("SGLT2 Inhibitors"[MeSH Terms] OR "sodium-glucose cotransporter 2 inhibitors" OR "canagliflozin" OR "dapagliflozin" OR "empagliflozin" OR "ertugliflozin" OR "ipragliflozin") AND ("Cardiovascular Diseases"[MeSH Terms] OR "cardiovascular outcomes" OR "heart failure" OR "myocardial infarction" OR "stroke") AND ("Neoplasms"[MeSH Terms] OR cancer OR "malignancy" OR "tumor" OR "oncology") AND ("Diabetes Mellitus, Type 2"[MeSH Terms] OR T2DM OR "type 2 diabetes")
Embase	('sglt2 inhibitor'/exp OR 'sodium glucose co transporter 2 inhibitor' OR canagliflozin OR dapagliflozin OR empagliflozin OR ertugliflozin OR ipragliflozin) AND ('cardiovascular disease'/exp OR 'cardiovascular outcome' OR 'heart failure' OR 'myocardial infarction' OR stroke) AND ('neoplasm'/exp OR cancer OR malignancy OR tumor OR oncology) AND ('type 2 diabetes mellitus'/exp OR T2DM OR 'type 2 diabetes')
Scopus	TITLE-ABS-KEY(("sglt2 inhibitor" OR "sodium-glucose cotransporter 2 inhibitor" OR canagliflozin OR dapagliflozin OR empagliflozin OR ertugliflozin OR ipragliflozin) AND ("cardiovascular outcome" OR "heart failure" OR "myocardial infarction" OR stroke) AND (cancer OR malignancy OR tumor OR neoplasm OR oncology) AND ("type 2 diabetes" OR T2DM))
Web of Science	TS=(("sglt2 inhibitor" OR "sodium-glucose cotransporter 2 inhibitor" OR canagliflozin OR dapagliflozin OR empagliflozin OR ertugliflozin OR ipragliflozin) AND ("cardiovascular outcome" OR "heart failure" OR "myocardial infarction" OR stroke) AND (cancer OR malignancy OR tumor OR neoplasm OR oncology) AND ("type 2 diabetes" OR T2DM))
